# Design of FLT3 Inhibitor - Gold Nanoparticle Conjugates as Potential Therapeutic Agents for the Treatment of Acute Myeloid Leukemia

**DOI:** 10.1186/s11671-015-1154-2

**Published:** 2015-12-01

**Authors:** Timea Simon, Ciprian Tomuleasa, Anca Bojan, Ioana Berindan-Neagoe, Sanda Boca, Simion Astilean

**Affiliations:** Nanobiophotonics and Laser Microspectroscopy Center, Interdisciplinary Research Institute on Bio-Nano-Sciences and Faculty of Physics, Babes-Bolyai University, T. Laurian 42, 400271 Cluj-Napoca, Romania; Department of Hematology, Ion Chiricuta Oncology Institute, Bulevardul 21 Decembrie 1918 Nr 73, 400124 Cluj-Napoca, Romania; Research Center for Functional Genomics and Translational Medicine, Iuliu Hatieganu University of Medicine and Pharmacy, Marinescu Street 23, 40015 Cluj-Napoca, Romania; Department of Experimental Therapeutics, The University of Texas MD Anderson Cancer Center, Houston, TX USA

**Keywords:** FLT3 inhibitors, Acute myeloid leukemia, Drug-loaded gold nanoparticles, Pluronic

## Abstract

**Background:**

Releasing drug molecules at the targeted location could increase the clinical outcome of a large number of anti-tumor treatments which require low systemic damage and low side effects. Nano-carriers of drugs show great potential for such task due to their capability of accumulating and releasing their payload specifically, at the tumor site.

**Results:**

FLT3 inhibitor - gold nanoparticle conjugates were fabricated to serve as vehicles for the delivery of anti-tumor drugs. Lestaurtinib, midostaurin, sorafenib, and quizartinib were selected among the FLT3 inhibitor drugs that are currently used in clinics for the treatment of acute myeloid leukemia. The drugs were loaded onto nanoparticle surface using a conjugation strategy based on hydrophobic-hydrophobic interactions with the Pluronic co-polymer used as nanoparticle surface coating. Optical absorption characterization of the particles in solution showed that FLT3 inhibitor-incorporated gold nanoparticles were uniformly distributed and chemically stable regardless of the drug content. Drug loading study revealed a high drug content in the case of midostaurin drug which also showed increased stability. Drug release test in simulated cancer cell conditions demonstrated more than 56 % release of the entrapped drug, a result that correlates well with the superior cytotoxicity of the nano-conjugates comparatively with the free drug.

**Conclusions:**

This is a pioneering study regarding the efficient loading of gold nanoparticles with selected FLT3 inhibitors. *In vitro* cytotoxicity assessment shows that FLT3-incorporated gold nanoparticles are promising candidates as vehicles for anti-tumor drugs and demonstrate superior therapeutic effect comparatively with the bare drugs.

**Electronic supplementary material:**

The online version of this article (doi:10.1186/s11671-015-1154-2) contains supplementary material, which is available to authorized users.

## Background

Acute myeloid leukemia (AML) is one of the most common and deadly leukemias worldwide [1–3]. Its prognosis is poor, and a large number of AML cases will relapse after initial healing. Clinicians currently use the FMS-like tyrosine kinase 3 (FLT3) inhibitors such as lestaurtinib, midostaurin, sorafenib, and quizartinib for AML therapy [4–6]. But the administration of these drugs systemically is associated with high toxicity and severe side effects [7–9]. Therefore, it would be helpful to refine the treatment accuracy by developing novel markers or drugs that could improve the therapeutic ratio and clinical outcome.

Nano-pharmacology is emerging as a new and highly promising solution for the management of leukemias. By designing specific delivery systems or nano-carriers, higher drug concentrations can be transported to the malignant cell [10–12]. Specifically related to acute myeloid leukemia, binding anti-FLT3 inhibitor drugs to nano-carriers might offer a new and exciting approach to an improved treatment. So far, no data has been published related to the possibility to bind gold nanostructures or any other nanoparticles to tyrosine kinase inhibitors (TKI), thus emphasizing the importance of the current manuscript in the advancements of nano-pharmacology. The only group that has so far attempted to use TKI transported through nano-carriers in order to improve the concept of personalized medicine was published by Ljubimova recently [13]. This group has used polymalic acid-based nano-biopolymers to treat Her2/positive breast cancer. But, no others have so far succeeded to improve the chemotherapy options using gold nanoparticle (GNP) conjugation to TKI for hematological malignancies, less alone AML. Directly related to the design and development of anti-leukemic drugs, this report presents a new class of GNP-FLT3 inhibitor nano-conjugates which are promising candidates for the treatment of AML. These nano-conjugates were fabricated to serve as vehicles for the delivery of FLT3 inhibitor drugs lestaurtinib (LST), midostaurin (MDS), sorafenib (SRF), and quizartinib (QZR). The drugs were loaded onto the surface of nanoparticles using a conjugation strategy based on hydrophobic-hydrophobic interactions with the Pluronic co-polymer used as nanoparticle surface coating. Optical absorption characterization of the nano-conjugates in solution showed that FLT3 inhibitor-incorporated GNP were uniformly distributed and chemically stable regardless of the drug content. Dynamic light scattering (DLS) measurements of the FLT3 inhibitor-loaded GNP revealed a relatively uniform size and morphology of drug-loaded particles. A drug loading study revealed an increased drug content in the case of MDS drug which also showed increased stability in phosphate buffer solution (PBS), in time. Release test was conducted at characteristic pH and glutathione level for cancer cells and demonstrated more than 56 % release of the entrapped drug, a result that correlates well with the measured superior cytotoxicity comparatively with the free drug or non-loaded nanoparticles.

This is a pioneering study regarding the efficient loading of GNP with four different FLT3 inhibitors and their characterization. *In vitro* cytotoxicity studies on two cell lines (OCI-AML3 and THP1) demonstrate that FLT3-loaded GNP are promising candidates as vehicles for anti-tumor drugs and show superior therapeutic effect than the freely administered drugs.

## Methods

### Materials

Hydrogen tetrachloroaurate (III) hydrate (HAuCl_4_^.^3H_2_O, 99.99 %), trisodium citrate (C_6_H_5_Na_3_O_7_), Pluronic F127 (powder, BioReagent, suitable for cell culture), lestaurtinib hydrate (CEP-701, >98 %), midostaurin hydrate (>98 %), and l-glutathione (cell culture tested, BioReagent, ≥98.0 %) were purchased from Sigma-Aldrich. Quizartinib (AC220, >99 %) was obtained from Seleckchem and Sorafenib (>99 %) from Santa Cruz Biotechnologies.

### Synthesis of Gold Nanoparticles

Citrate-capped spherical gold nanoparticles (GNP) were synthesized by the aqueous reduction of HAuCl_4_ with trisodium citrate according to the Turkevich-Frens method [14]. Briefly, 100 ml of 1 mM HAuCl_4_^.^3H_2_O was boiled, and a solution of 38.8 mM sodium citrate (10 mL) was quickly added under vigorous stirring. During the boiling process, the solution changed its color from yellow to intense burgundy-red. Then, the solution was removed from the heat, and the stirring process continued for another 10–15 min.

### Design of FLT3 Inhibitor - Gold Nanoparticle Conjugates

FLT3 inhibitors were dissolved in DMSO at a final concentration of 1 mg/ml. Forty microliters of these solutions was added to 1 ml of GNP and incubated for a specific time in the case of each drug. The incubation time was determined in such a way to allow interaction with GNP but to not induce aggregation (90 s for MDS, 60 s for LST, 30 s for SRF, and QZR was incubated together with Pluronic). Then, to capture the hydrophobic molecules onto the surface of GNP and to stabilize the obtained conjugates, Pluronic F127 was added to the colloidal solution at a final concentration of 0.3 mM. After 1 h incubation, the formed nano-conjugates were purified from any unbound drug and polymer by centrifugation at 12,000 rpm for 20 min and resuspended in ultrapure water for further measurements or in PBS for stability tests and *in vitro* assays.

### Loading Efficiency of FLT3 Inhibitors onto GNP

The amount of drugs loaded to GNP-Pl was calculated by measuring the drug content of the supernatant after purification of the particles by centrifugation. The loading efficiency was then calculated using the following formula:

$$ \%\mathrm{Loading}\kern0.5em \mathrm{efficiency}=\frac{T_{\mathrm{drug}}-{S}_{\mathrm{drug}}}{T_{\mathrm{drug}}}\ast 100 $$

where *T*_drug_ is the total amount of drug added and *S*_drug_ represents the amount of drug in supernatant. Drug loading was calculated by estimating the free drug content of the supernatant remained after centrifugation of the samples. The free drug concentrations in the supernatant were determined from the calibration curve based on Beer-Lambert law by linear interpolation of the readings of supernatant absorbances.

### GSH-Mediated Release of MDS

To investigate MDS release from gold nanoparticles, 150 μl of GNP-MDS-Pl dispersed in PBS was mixed with 350 μl acetate buffer at pH 4.6 containing 10 mM glutathione, and the mixture was maintained at constant 37 °C for 24 h. For control measurements, the same amount of nanoparticles was dispersed in PBS and kept in the dark at 4 °C. At different intervals of time (0, 6, and 24 h), both control and GSH mixtures were centrifuged, and the amount of MDS released into the supernatant was calculated based on UV-Vis absorption measurements.

### Cell Culture Protocols

All cells were grown at 37 °C in a humidified atmosphere of 95 % air and 5 % carbon dioxide (CO_2_). Cell passage and culture were carried out as previously described [15, 16]. OCI-AML3 cell line was cultured in 80 % alpha-MEM + 20 % fetal bovine serum (FBS) (both from Sigma-Aldrich), the cells being initially isolated from the peripheral blood of a 57-year-old man with acute myeloid leukemia (AML FAB M4) at diagnosis in 1987. Cells carry an NPM1 gene mutation (type A) and the DNMT3A R882C mutation [17]. This cell line was kindly gifted by Professor George Calin, MD, PhD (Department of Experimental Therapeutics, The University of Texas MD Anderson Cancer Center, Houston, TX, USA). THP1 cells were cultured using ATCC-formulated RPMI-1640 Medium (ATCC, USA), being isolated from an acute monocytic leukemia Japanese patient in 1980 [18]. The cell line was kindly gifted by Associate Professor Mihnea Zdrenghea, MD, PhD (Department of Hematology, Iuliu Hatieganu University of Medicine and Pharmacy/Ion Chiricuta Oncology Institute, Cluj Napoca, Romania).

### Cell Counting

1.5 × 10^3^ cells were plated in 24-well plates (day 0), treated with the various drug combinations after 24 h, and counted at days 1, 2, 4, 7, 10, and 14 by using both a hemocytometer and a Leica S80 inverted phase contrast microscope, as well as the Countess Automated Cell Counter (Invitrogen).

### MTT Assay

Cell survival was assessed using the 3-(4,5-dimethylthiazol-2-yl)-2,5-diphenyltetrazolium bromide (MTT) assay. Cells in monolayer culture were cultivated at sub-confluence before being washed twice with PBS. Cells were then resuspended in culture medium with FBS, counted, and plated in 100 μl media at 15 × 10^3^ cells/well in 96-well microliter plates. After 24 h, the cells were washed and treated with drugs/nanoparticles. The final drug concentrations used for the *in vitro* assays were established after the determination of the IC50 after extensive pilot projects (data not shown in this paper) have been completed and having established the best concentrations of drugs that should be used. The cell culture medium was changed every 3 days with fresh medium. Every time we changed the medium with a fresh one, we centrifuged the cells and re-plated them in different cell culture flasks. After the re-plating, we added freshly made culture media, supplemented with the investigated drugs and nanoparticles at the same concentration as in the initial plating.

FLT3 drug-loaded GNP were compared with the corresponding conventional TKI at identical concentrations. Absorbance of the MTT was measured at 492 nm using a BioTek Synergy.

### Statistical Analysis

The statistical analysis was performed using R (R Development core team, USA) and GraphPad Prism 5.0 (GraphPad Software INC, CA, USA). The obtained data was first examined for normality of distribution using the Shapiro-Wilk test. The distribution of all the obtained data was Gaussian, thus it was analyzed using a parametric test (two-way ANOVA with Tukey’s post-test). The differences were considered significant when *p* < 0.05.

### Equipment

Absorption spectra were recorded using a Jasco V-670 UV-Vis-NIR spectrometer with a slit width of 2 and 1 nm spectral resolution. Particle size distribution and zeta potential were measured by a Zetasizer NanoZS90 instrument (Malvern Instruments). Analysis was performed at a scattering angle of 90° and temperature of 25 °C. Dark field images of OCI-AML3 and THP1 cells incubated with GNP-MDS-Pl were acquired using an inverted Zeiss Axio Observer Z1 microscope. To capture the cells, a few microliters of cell suspension were dropped onto an Ibidi μ-Dish and enclosed with a cover slip. A 100-W halogen lamp was used for illumination which was focused on the sample using a high numerical immersion condenser (NA = 1.4), and the scattered light was collected by an LD Plan-Neofluar ×20 objective (NA = 0.4, Zeiss).

## Results and Discussion

### Synthesis and Characterization of FLT3 Inhibitor - Gold Nanoparticle conjugates

**Fig. 1:**
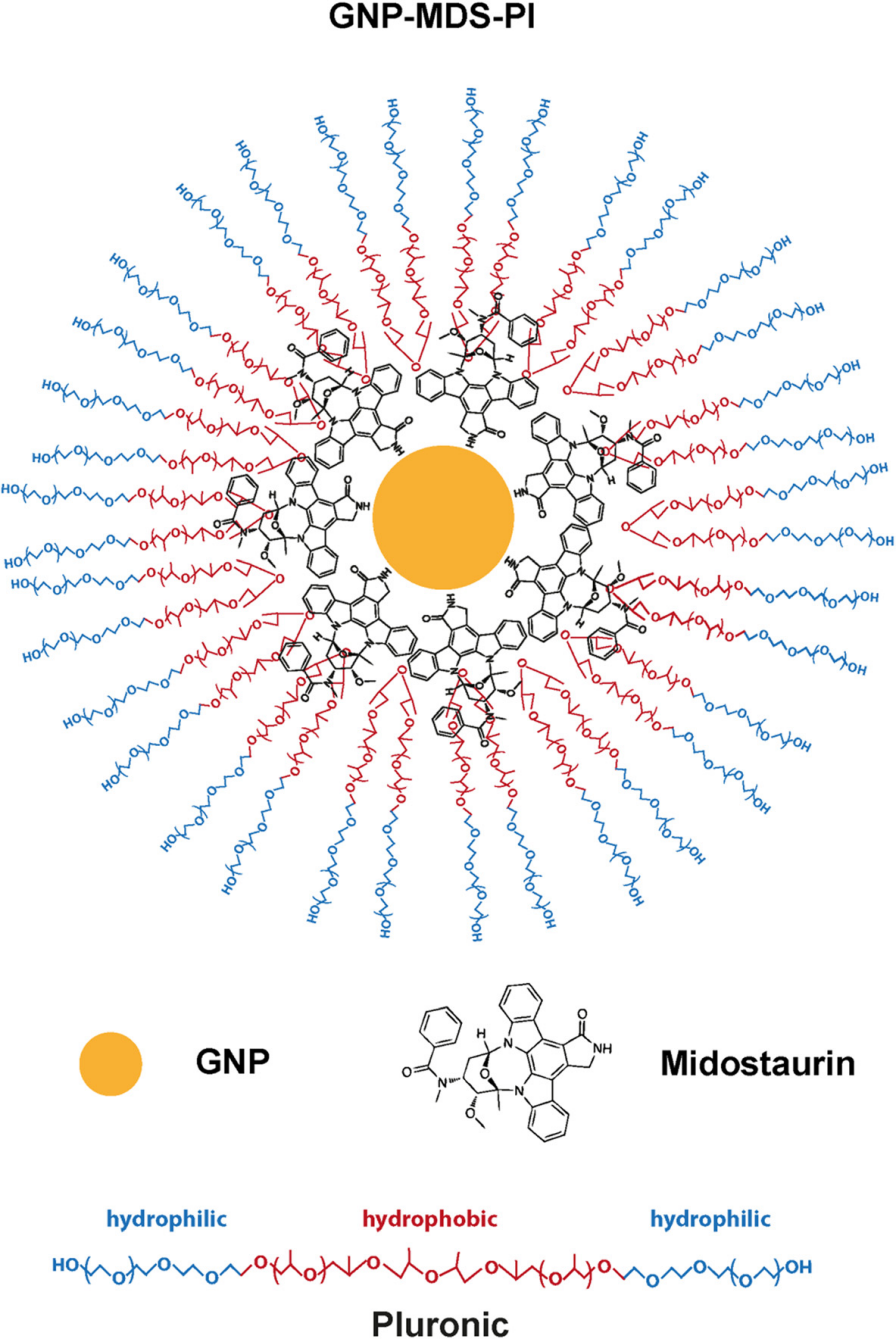
Schematization of gold nanoparticle loading with midostaurin drug and stabilization with Pluronic

The strategy employed for conjugating FLT3 inhibitors onto GNP implies the using of Pluronic F127 block co-polymer as overall coating surface. Pluronic is an amphiphilic polymer composed of hydrophobic poly(propylene oxide) chains at the core and hydrophilic poly(ethylene oxide) chains at the ends arranged in a A-B-A structure (Fig. 1). Due to its amphiphilic nature and temperature-dependent micellization characteristics, Pluronic can be exploited for water-solubilization of hydrophobic compounds and as drug carriers in delivery applications [19, 20]. Grafting a polymeric coating onto GNP surface has the role of mediating the binding of drug molecules and, equally important, to provide stability of the particles in biological media. By interfacing nanoparticles with the polymer above, we aimed to find the optimal type of nano-platform which provides the maximum loading efficiency of the drug molecules onto GNP and the highest stability in biologically relevant media. The conjugation strategy relies on the direct attachment of FLT3 drugs onto the surface of GNP followed by their subsequent encapsulation in Pluronic as schematized in Fig. 1.

GNP present a strong absorption band in the visible region originating from the collective oscillation of free electrons under light excitation, known as localized surface plasmon resonance (LSPR). The intensity and wavelength of the LSPR band depends on a variety of factors which modify the electron charge density on the particle surface, such as particle size and shape, structure, composition, and the dielectric constant of the surrounding medium [21, 22]. Due to the high sensitivity of the LSPR to the local refractive index of the environment surrounding GNP surface, the loading of the drugs onto nanoparticle surface can be efficiently monitored.

**Fig. 2:**
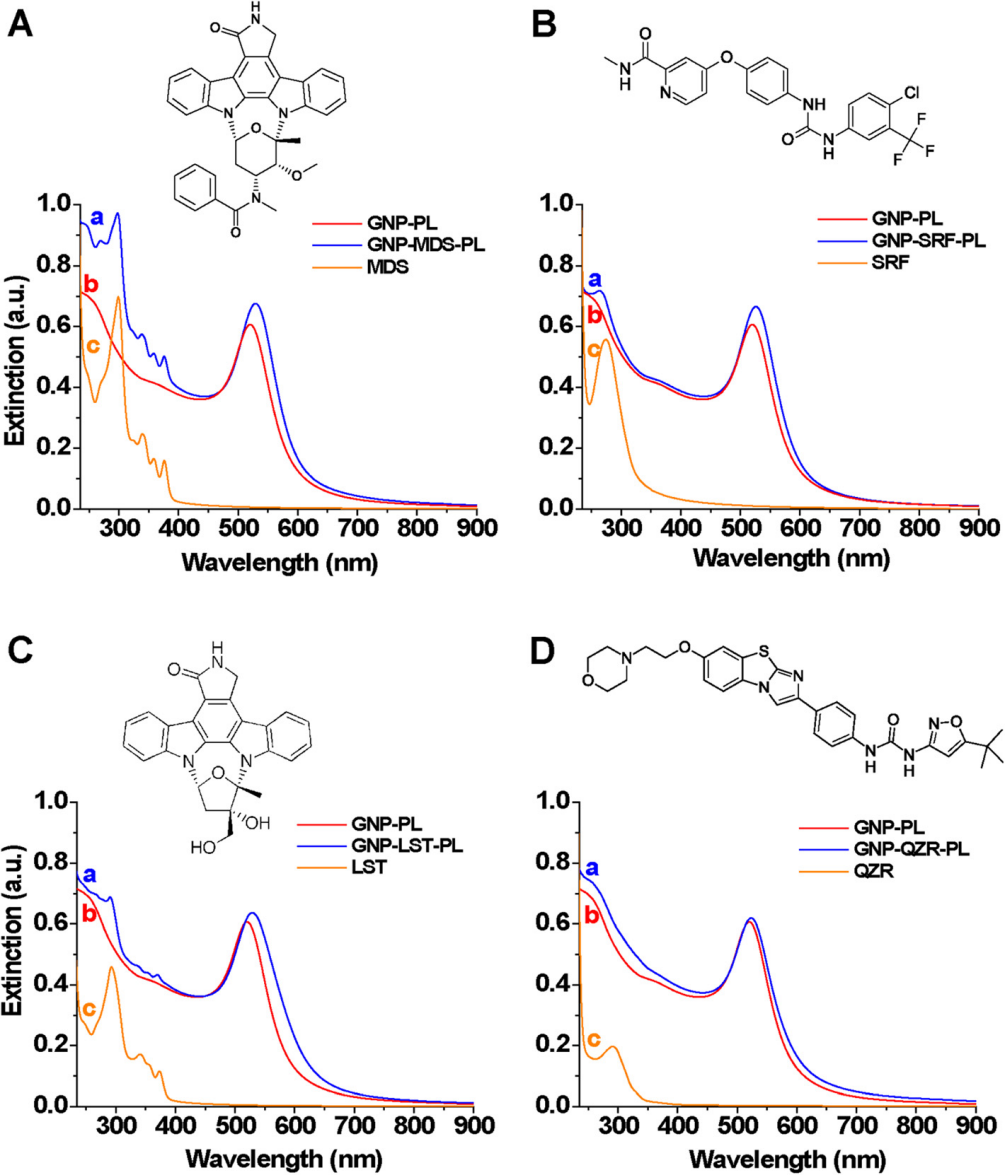
UV-Vis extinction spectra measured in water of FLT3-inhibitor conjugated GNP-Pl (**a**), control GNP-Pl (**b**), and absorption spectra of FLT3-inhibitors (**c**). (**d**) A-MDS, B-SRF, C-LST, D-QZR. Corresponding chemical structures of the investigated drugs are illustrated in the upper side of the figures

Therefore, we recorded the LSPR spectra of GNP in water before and after interaction with the FLT3 inhibitor drugs (MDS, SRF, LST, and QZR) and encapsulation in Pluronic (Fig. 2). UV-Vis absorption spectra of free drugs (in solution) were also recorded, for reference. In all the cases, the addition of the drug molecules to the solutions of nanoparticles induced a red-shift of their LSPR maximum, a result which can be considered as a first proof of nanoparticle loading. The spectral positions of the absorption maxima are also summarized in Table 1. The differences in the magnitude of the LSPR shift (from a value of 3 nm in the case of QZR to 9 nm in the case of MDS) can be ascribed to the variation of the refractive index in the vicinity of GNP as a consequence of the different molecular species and the amount of drug loaded. Besides the plasmonic bands in the visible region, each spectrum of FLT3 drug-GNP nano-conjugate presents spectral features also in the UV domain, which originate from the electronic absorption of the encapsulated FLT3 inhibitor molecules (Fig. 2). In our opinion, a N-Au bonding between drugs and nanoparticles is expected. For instance, MDS can bind through its oxindole group which was found to attach to gold nanoparticles [23, 24]. In the presence of Pluronic, the hydrophobic PPO blocks of Pluronic encaged the hydrophobic drug-nanoparticle conjugates while the hydrophilic PEO chains are extended through the aqueous phase ascertaining the solubility of nanoparticles in water.

**Table 1:** Size of FLT3 inhibitor conjugated GNP measured by DLS and PdI, their zeta potential together with zeta deviation, LSPR position, and calculated drug loading efficiency

Sample	Zeta potential (mV)	Zeta deviation (mV)	Diameter (nm)	Polydispersity index (PdI)	LSPR position (nm)	Loading efficiency (%)
GNP	−44 ± 2	5 ± 1	17 ± 2	0.45	519	–
GNP-PL	−25 ± 2	10 ± 1	30 ± 1	0.54	520	–
GNP-MDS-PL	−20 ± 1	9.8 ± 1	58 ± 2	0.46	529	80
GNP-SRF-PL	−17.5 ± 0 .5	7.8 ± 3	48 ± 1	0.52	526	10
GNP-QZR-PL	−5 ± 0.7	4 ± 0.2	58 ± 2	0.53	523	8
GNP-LST-PL	−10 ± 0.5	8.5 ± 0.3	44 ± 2	0.95	527	10

For each set of GNP-FLT3 inhibitor nano-conjugate, the loading efficiency was calculated based on the UV-Vis measurements as described in the experimental section (Table 1). The most efficient drug loading was found to be 80 % in the case of MDS, while conjugation with the other three drugs determined relatively low loading values (5–10 %). It is noteworthy that the shape of the LSPR band remained unaffected in all of the four cases, which indicates that the particles remained individual in suspension and the conjugation with the drugs did not cause any aggregation.

To further ascertain the formation of FLT3 drug-GNP conjugates and get supplementary information regarding the conjugation, DLS and zeta potential measurements were performed. These techniques, which provide information about hydrodynamic diameter of the nanoparticles in colloidal solutions and their surface charge in suspension, are ideal methods to assess the loading of drugs [25]. It is worth to mention that the diameters measured by DLS give fairly higher values as measured by transmission electron microscopy (TEM), since it includes also the surface coating of nanoparticles. For instance, bare GNP having a physical diameter of ~12 nm measured by TEM give a hydrodynamic diameter of 17 nm by DLS. After encapsulation in Pluronic, the hydrodynamic diameter of GNP increases to 30 nm. Compared to the drug-free nanoparticles (GNP-Pl), the obtained nano-conjugates have significantly higher hydrodynamic diameters (Table 1), which cannot be solely attributed to the amount of drug loaded. It is known that Pluronic co-polymers adsorb in different organizations onto surfaces with different hydrophobicity [26]. When adsorbing onto a hydrophobic surface in aqueous solution, Pluronic has a “brush”-like organization with the hydrophobic PPO block grafted to the surface while the hydrophilic PEO chains extend into the aqueous phase and cover the surface with a PEO layer. On the contrary, for a hydrophilic surface, Pluronic tends to attach to the surface more loosely by the two PEO segments and form a less “pancake” structure [27]. As citrate-capped gold nanoparticles own a hydrophilic surface, we can assume such pancake organization of Pluronic when stabilized without drug loading. On the other hand, after association with the hydrophobic drugs, the surface of nanoparticles became more or less hydrophobic depending on the surface coverage by drugs; therefore, a brush-like adsorption is more probable. Moreover, the amount of Pluronic adsorbed on a hydrophilic surface is generally lower than that on a hydrophobic surface. We can attribute the large difference in hydrodynamic diameter obtained between drug-loaded and drug-free GNP-Pl to the different amount of Pluronic adsorbed and to the different organization at the surface of nanoparticles in function of their hydrophobicity. Compared in between the four drugs, the highest increase was observed for the GNP-MDS-Pl conjugate which, in the one hand, is in very good concordance with both the high loading efficiency and the largest LSPR red-shift observed in the optical spectrum. On the other hand, owing to the high concentration of MDS loaded, we can assume that this type of particles have the most hydrophobic surface after conjugation. We do not exclude a combined polymer organization in case of nanoparticles with lower drug content.

Moreover, the surface charge of the nanoparticles also modifies as consequence of the interaction with the FLT3 inhibitors and Pluronic (Table 1).

As the stability of the particles is a critical issue for any use in biological media, the evolution of the extinction spectra of the prepared nano-conjugates were monitored over time in PBS (Additional file 1: Figure SF1). GNP-MDS-Pl proved to be very stable after 1 week storage in PBS, without any modification of the plasmonic band. Contrarily, in the case of GNP-SRF-Pl, the appearance of a second plasmonic maximum at longer wavelengths (~640 nm) indicates the aggregation/agglomeration of the nanoparticles, most probably organization in chain-like structures [28]. Such kind of chain formation was also observed in the case of GNP conjugation with MDS if no Pluronic was added to stabilize the nano-conjugates. Because of the hydrophobicity of these drug molecules, in lack of a surface coating which can provide dispersability in hydrophilic medium or in case of insufficient surface coverage, the conjugated GNP tend to aggregate or self-assembly. A slight aggregation also occurs in the case of GNP-LST-Pl and GNP-QZR-Pl after 1 week storage in PBS, indicated by the broadening of the plasmonic band of the nanoparticles. However, they are very similar in their chemical structure, due to the two OH groups (see chemical structure in Fig. 2). LST is more hydrophilic than MDS, which weakens considerably the interaction with Pluronic, thus resulting insufficient stability. These results sustain our presumption regarding the different types of adsorption of Pluronic onto the surface of nanoparticles depending on their hydrophobicity.

### Drug Release Test

**Fig. 3:**
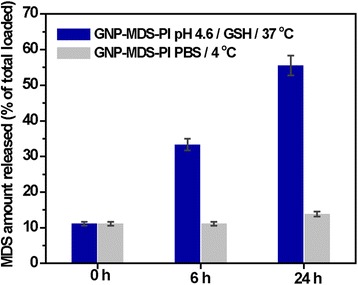
GSH and pH-mediated drug release by GNP-MDS-Pl

Since the most efficient FLT3 drug loading was found for the GNP-MDS-Pl which were also the most stable in physiological medium, in the following, we limited our investigations to this type of nano-conjugates. The release of the drug payload from nanoparticles was investigated under simulated biological conditions of cancer cells, i.e., at 37 °C at a typical intracellular glutathione (GSH) level (10 mM) and pH 4.6. On the one hand, this release strategy relies on the significant difference in intracellular and extracellular GSH concentration (1-10 mM intracellular [29] and 2 μΜ in red blood plasma [30]). GSH has been widely utilized as an external trigger for drug releases, due to the strong affinity of the thiol ligand to the gold surface, able to counteract with other types of interactions at molecular level and to take place in exchange reactions that can result the dissociation of drug molecules from the nanoparticles, which will consequently be released. As the N–Au bond is relatively weak (6 kcal/mol) compared to the S–Au bond (47 kcal/mol) [31], one can expect that GSH can replace the more loosely bound drug molecules at the surface of gold nanoparticles. Such results were also obtained by Mira Kim et al., who demonstrated that the anti-cancer drug topotecan adsorbed onto the gold nanoparticle surface through its nitrogen atoms was detached via external glutathione both *in vitro* and *in vivo* [32]. On the other hand, the acidic environment in endosomal and lysosomal compartments (4.5 < pH < 5) of cancer cells [33] can also trigger the release of drugs from pH responsive polymers such as Pluronic [34, 35]. In Fig. 3, the release is expressed in wt% of the total amount of MDS attached to the nanoparticles calculated using an UV-Vis absorption calibration curve. GNP-MDS-Pl subjected to simulated biological conditions (10 mM GSH, pH 4.6, and temperature of 37 °C) show a significant drug release after 6 h of incubation (33 %), which increased up to 56 % release of the total drug content in 24 h. For control sample, GNP-MDS-Pl in PBS kept at 4 °C, only a release of 10 % can be observed. UV-Vis absorption spectra of the supernatants and the nanoparticles after each centrifugation step (0, 6, and 24 h) are shown in Additional file 1: Figure SF2 and SF3. After releasing the drug, the plasmonic band of nano-conjugates suffered a significant broadening which can be attributed to aggregated or interconnected nanoparticles as a consequence of elimination of the stabilizing Pluronic layer. The decrease of the absorption band of MDS at 295 nm corroborates well the amount of drug released and eliminated by the supernatant.

### Dark Field Imaging of MDS-Gold Nanoparticle Conjugates Inside Cells

**Fig. 4:**
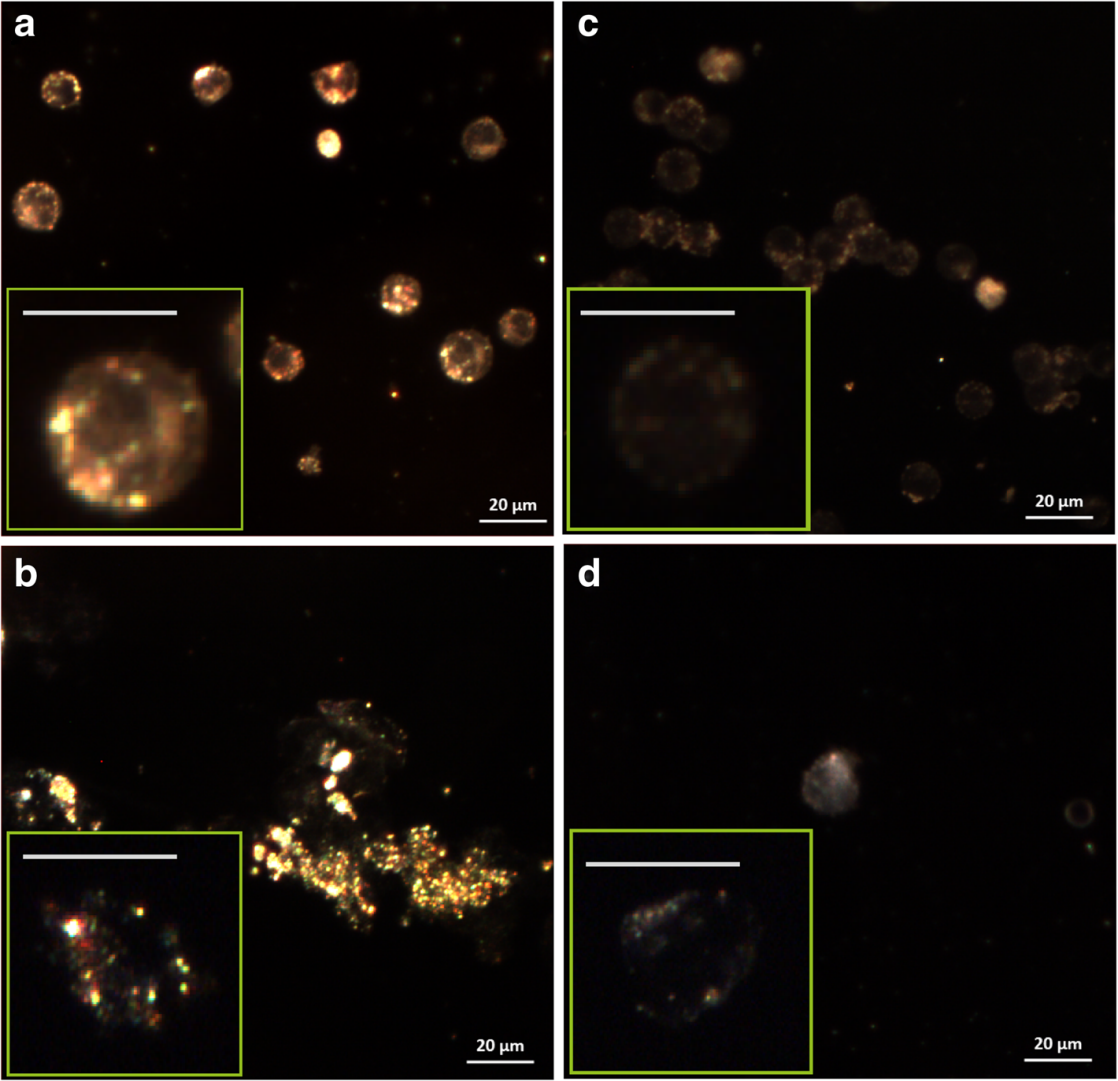
Dark field image of OCI-AML3 (**a**) and THP1 (**b**) cells in the presence of GNP-MDS-Pl nano-conjugates. Dark field image of OCI-AML3 (**c**) and THP1 (**d**) cells (without nanoparticles). The *insets* show magnified images of cells; *scale bars* in insets represent 10 μm

The nanoparticle internalization by cells was analyzed by dark field microscopy. Figure 4 presents the dark field image of OCI-AML3 cells (A) and THP-1 (B) after 24 h incubation with GNP-MDS-Pl. The nanoparticles can be clearly visualized inside cells as diffraction-limited sparkling (bright orange to reddish) dots due to the effective light scattering at their SPR. GNP are usually uptaken by cells via the endocytotic mechanism and accumulated in lysosomes [36]. Zoomed images from the inset of Fig. 4a, b reveal an extranuclear localization of our nanoparticles inside both cell lines, the nucleus being clearly delimited as a dark region. The variation in color of the nanoparticles is related to various degrees of agglomeration of the nanoparticles inside cellular vesicles. No similar scattering pattern can be observed in the control sample, without nanoparticles, which exhibit light scattering by cellular structures of various optical densities (Fig. 4b, d).

### *In Vitro* Evaluation of MDS-Gold Nanoparticle Conjugates

**Fig. 5:**
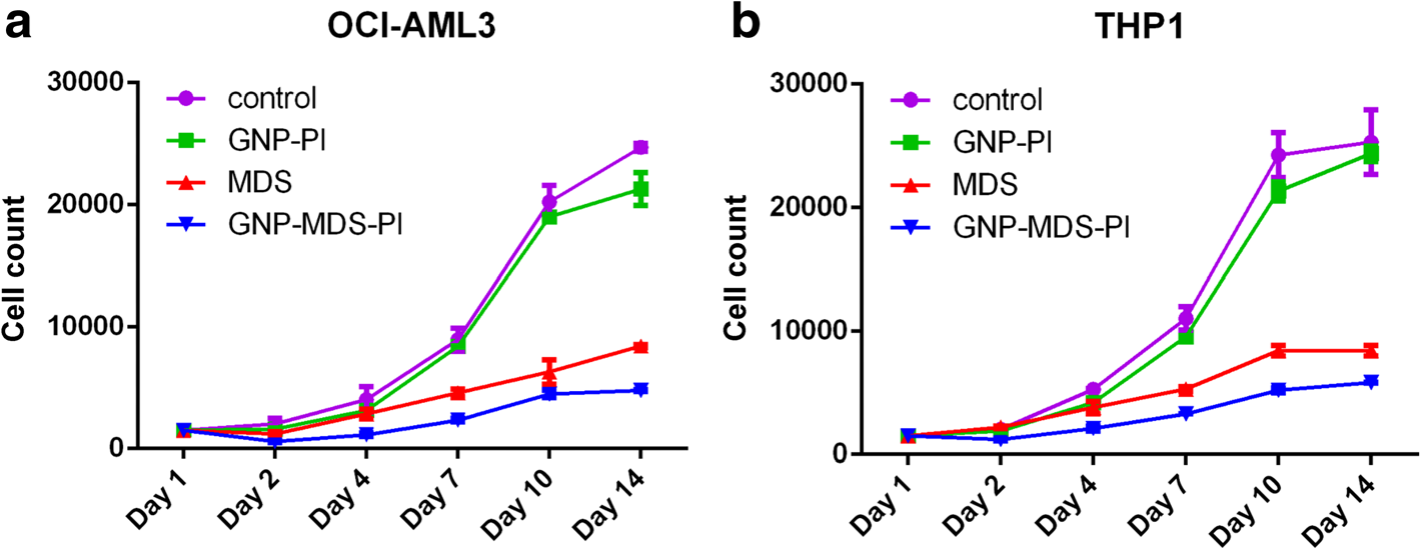
Survival curves for both OCI-AML3 cell line (**a**) and THP1 cell line (**b**)

Current clinical data used MDS in combination with other anti-cancer drugs such as 5-azacytidine [37], but this is accompanied by further toxicity and a decreased quality of life for the leukemia patient [38]. Chemotherapy-refractory or relapsed AML patients have a dismal prognosis, and most of the times they do not reach the curative stem cell transplantation. Thus, we must use drugs that have already been developed and further enhance their effect through a more increased cancer cell penetration and a decreased efflux of the drug. The newly synthesized nanostructures were evaluated *in vitro* in order to assess their potential efficacy in AML chemotherapy. The leukemia cell proliferation was evaluated using both standard cell counting, as well as the spectrophotometer-based assay MTT. Figure 5a and b shows the survival curves for both OCI-AML3 cell line and THP1 cell line, respectively. We can clearly see that the TKI have a direct anti-leukemia effect, further enhanced by the newly described drug, after being loaded to GNP. We have chosen to enhance the effect of MDS, as the drug alone has yet to reach its expectations, as also confirmed by the clinical data [39, 40].

**Fig. 6:**
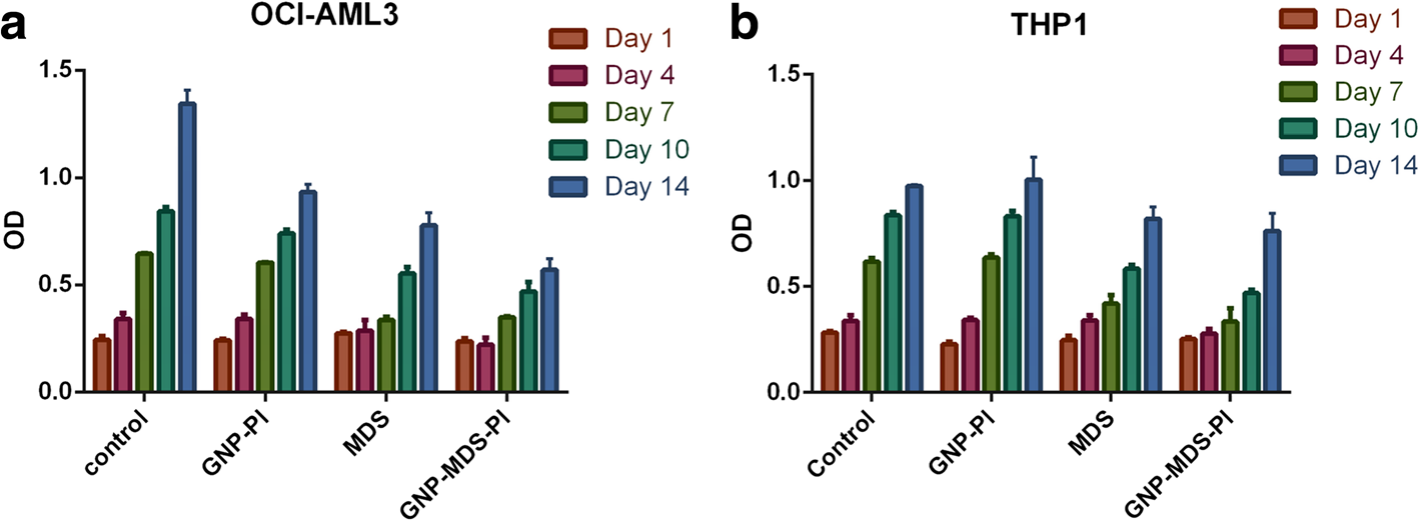
MTT cell proliferation assay for the OCI-AML3 (**a**) and THP1 cell lines (**b**)

The classic cell proliferation assay data was later on confirmed using the MTT assay, as seen in Fig. 6a (for the OCI-AML3 cell line) and 6b (for the THP1 cell line). The data obtained using the proliferation assays are statistically significant, as shown in the Additional file 1: Tables SF1 and SF2.

## Conclusions

This paper presents the first demonstration of GNP loading with selected FLT3 inhibitor drugs (midostaurin, sorafenib, quizartinib, and lestaurtinib) and their assessment as therapeutic agents against AML, inside living cells. The efficient formation of FLT3-GNP bioconjugate complex encapsulated in Pluronic was demonstrated by their systematic characterization with various techniques, including optical spectroscopy, DLS, and zeta potential in solution. The loading efficiency was calculated for each of the drug nano-conjugate and was found to be highest in the case of MDS-loaded nanoparticles, which also proved increased robustness and stability in time. Drug release tests were conducted on GNP-MDS-Pl and showed that more than half of the total drug amount was released under simulated intracellular tumor cell conditions, a result which corroborates with an increased therapeutic effect of the nano-conjugates when compared with the free drug.

## Additional file

Additional file 1:
**Figure SF1.** Nanoparticle stability test by optical spectroscopy. **Figure SF2.** Absorption spectra of supernatants from drug-release assay. **Figure SF3.** Optical response of GNP-MDS-Pl after release. **Table SF1.** Statistical analysis for the cell proliferation for OCI-AML3 cell line. **Table SF2.** Statistical analysis for the cell proliferation for THP1cell line.

## Abbreviations

AML: acute myeloid leukemia
FLT3: FMS-like tyrosine kinase 3
GNP: gold nanoparticles
GSH: glutathione
LSPR: localized surface plasmon resonance
LST: lestaurtinib
MDS: midostaurin
QZR: quizartinib
SRF: sorafenib
TKI: tyrosine kinase inhibitors
