# Supplementary material for: Design of FLT3 Inhibitor - Gold Nanoparticle Conjugates as Potential Therapeutic Agents for the Treatment of Acute Myeloid Leukemia
Source: Nanoscale Res Lett. 2015 Dec 1;10:466. doi: 10.1186/s11671-015-1154-2 (PMC4666845; doi:10.1186/s11671-015-1154-2)
Supplement: Additional file 1: — Figure SF1. Nanoparticle stability test by optical spectroscopy. Figure SF2. Absorption spectra of supernatants from drug-release assay. Figure SF3. Optical response of GNP-MDS-Pl after release. Table SF1. Statistical analysis for the cell proliferation for OCI-AML3 cell line. Table SF2. Statistical analysis for the cell proliferation for THP1cell line. [file 11671_2015_1154_MOESM1_ESM.pdf]

Additional file

Design of FLT3 inhibitor-gold nanoparticle conjugates as potential therapeutic agents for the treatment of acute myeloid leukemia

Timea Simon[1], Ciprian Tomuleasa[2,3], Anca Bojan[2], Ioana Berindan-Neagoe[3,4], Sanda Boca[1], Simion Astilean[1,*]

[1] Nanobiophotonics and Laser Microspectroscopy Center, Interdisciplinary Research Institute on Bio-Nano-Sciences and Faculty of Physics, Babes-Bolyai University, T. Laurian 42, 400271 Cluj-Napoca, Romania

[2] Department of Hematology, Ion Chiricuta Oncology Institute, Bulevardul 21 Decembrie 1918 Nr 73, 400124, Cluj-Napoca, Romania

[3] Research Center for Functional Genomics and Translational Medicine, Iuliu Hatieganu University of Medicine and Pharmacy, Marinescu Street 23, 40015, Cluj-Napoca, Romania.

[4] Department of Experimental Therapeutics, The University of Texas MD Anderson Cancer Center, Houston, TX, United States of America

simion.astilean@phys.ubbcluj.ro (*corresponding author)

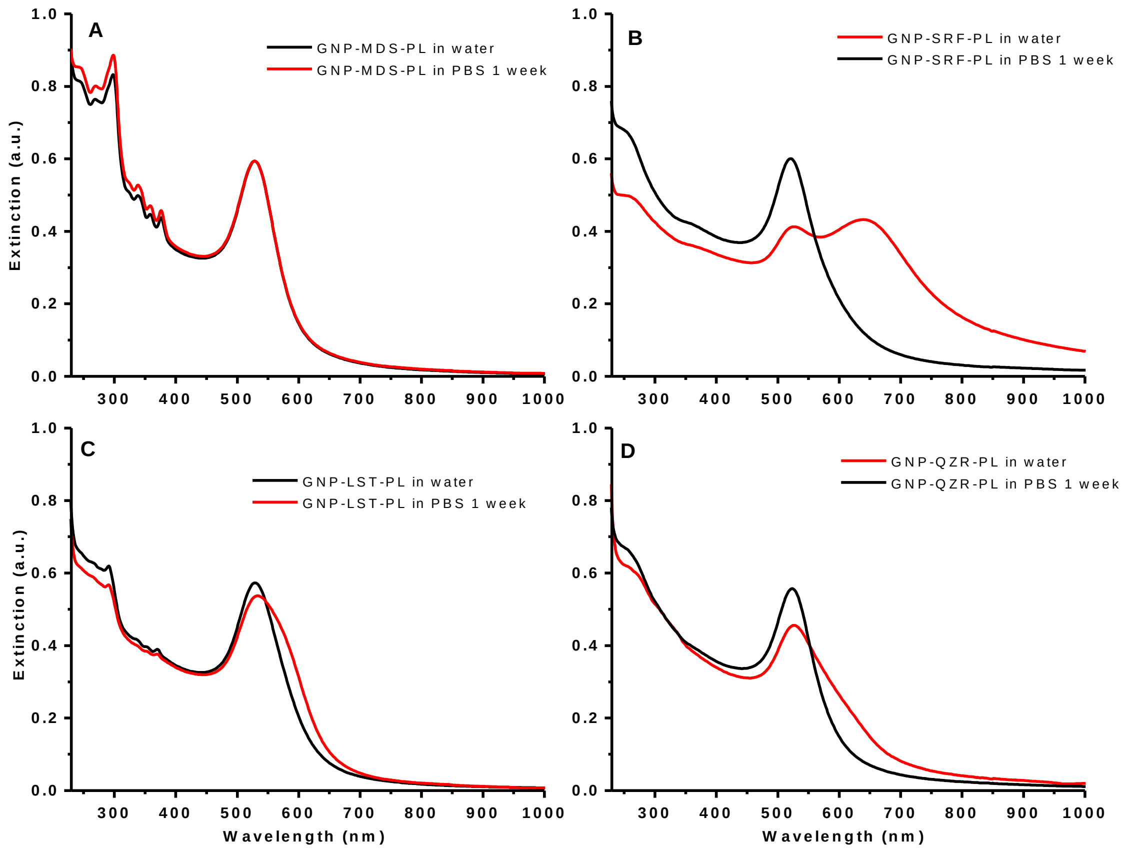

Figure SF1. Nanoparticle stability test by optical spectroscopy. UV-Vis absorption spectra of FLT3 inhibitor drugs-conjugated GNP (A-MDS; B-SRF; C-LST; D-QZR) in water (black lines) and after 1 week storage PBS (red lines).

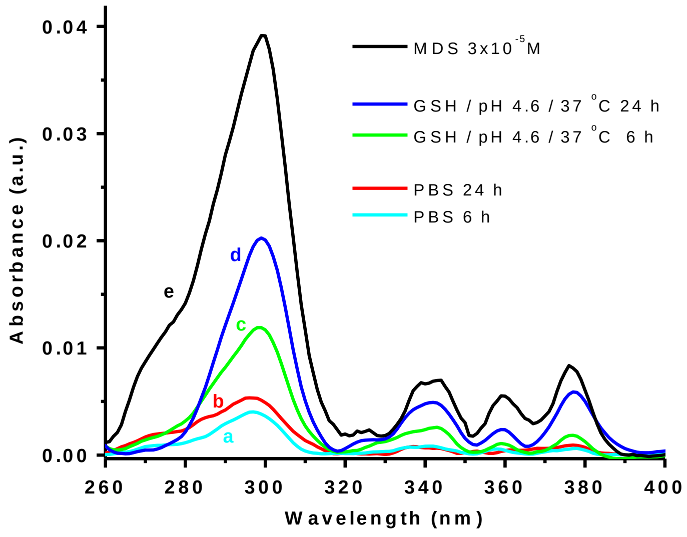

Figure SF2 Absorption spectra of supernatants from drug-release assay. Background corrected UV-Vis absorption spectra of the supernatant from GNP-MDS-Pl in control conditions (PBS, 4 ºC) after 6 hours (**a**); in control conditions (PBS, 4 ºC) after 24 hours (**b**); in release conditions (GSH, pH 4.6, 37 ºC) after 6 hours (**c**); release conditions (GSH, pH 4.6, 37 ºC) after 24 hours (**d**). Spectrum **e** represents the absorption spectrum of 30 μM MDS, corresponding to the total drug amount loaded onto GNP.

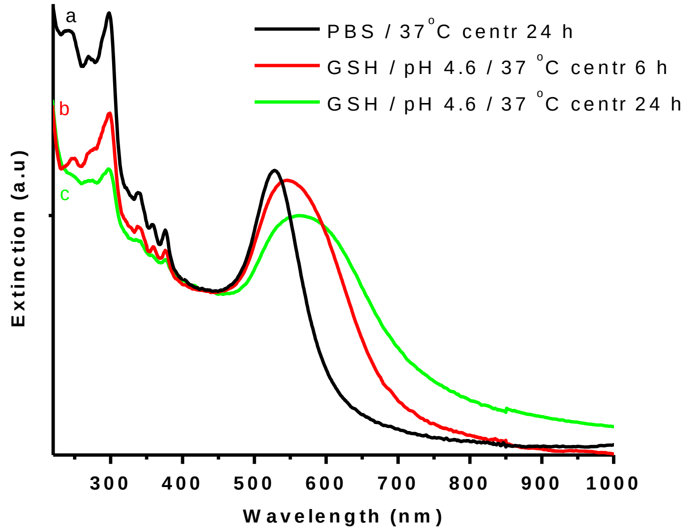

Figure SF3. Optical response of GNP-MDS-Pl after release. UV-Vis absorption spectra of GNP-MDS-Pl in PBS at 4 ^{0}C (**a**), centrifuged and resuspended in PBS after 6 h release test (**b**) and after 24 h release test (**c**).

Table SF1. Statistical analysis for the cell proliferation for OCI-AML3 cell line

Two-way ANOVA				
Source of Variation	% of total variation	P value		
Interaction	12,10	0,0006		
Column Factor	61,82	P<0.0001		
Row Factor	15,67	P<0.0001		
Source of Variation	P value summary	Significant?		
Interaction	***	Yes		
Column Factor	***	Yes		
Row Factor	***	Yes		
Source of Variation	Df	Sum-of-squares	Mean square	F
Interaction	12	0,7028	0,05857	3,874
Column Factor	4	3,592	0,8980	59,40
Row Factor	3	0,9107	0,3036	20,08
Residual	40	0,6047	0,01512	
Number of missing values	0			

Table SF2. Statistical analysis for the cell proliferation for THP1cell line

Two-way ANOVA				
Source of Variation	% of total variation	P value		
Interaction	5,67	0,0003		
Column Factor	79,84	P<0.0001		
Row Factor	9,93	P<0.0001		
Source of Variation	P value summary	Significant?		
Interaction	***	Yes		
Column Factor	***	Yes		
Row Factor	***	Yes		
Source of Variation	Df	Sum-of-squares	Mean square	F
Interaction	12	0,2336	0,01947	4,134
Column Factor	4	3,292	0,8230	174,7
Row Factor	3	0,4094	0,1365	28,98
Residual	40	0,1884	0,004710	
Number of missing values	0			
